# Therapeutic misalignment averted by clonal evolutionary evidence: molecular confirmation of hepatic metastasis in SMARCA4-deficient non-small cell lung cancer initially misdiagnosed as resectable cholangiocarcinoma

**DOI:** 10.3389/fonc.2026.1743908

**Published:** 2026-03-11

**Authors:** Ruirui Fan, Yanyan Zhan, Junrong Yan, Jie Gao

**Affiliations:** 1 Department of Anatomia Pathology, The Islands Healthcare Complex-Macao Medical Center of Peking Union Medical College Hospital, Macao, China; 2 Medical Department, Nanjing Geneseeq Technology Inc., Nanjing, China; 3 Department of Pathology, Xiamen Humanity Hospital, Xiamen, China

**Keywords:** clonal evolution analysis, hepatic metastasis, molecular profiling, pathological diagnosis, SMARCA4-deficient non-small cell lung cancer

## Abstract

**Objectives:**

*SMARCA4*-deficient non-small cell lung cancer (*SMARCA4*-dNSCLC) exhibits significant histomorphological diversity and intertumoral heterogeneity. Immunophenotypically, it often lacks lineage-specific markers, making diagnosis challenging, especially in cases of hepatic metastasis. Currently, no reliable method exists to distinguish these metastases from primary hepatic malignancies based on conventional pathology. Therefore, molecular profiling of somatic alterations combined with clonal evolutionary analysis is critical for accurate diagnosis.

**Methods:**

Histomorphological and immunophenotypic differences between pulmonary and hepatic lesions were assessed via hematoxylin and eosin staining and immunohistochemistry. Somatic mutational profiles were analyzed using next-generation sequencing, followed by clonal evolutionary analysis to confirm primary lung malignancy and hepatic metastasis.

**Results:**

This study presents a case of *SMARCA4*-dNSCLC exhibiting notable histomorphological and immunophenotypic divergence between the primary lung tumor and its hepatic metastasis. This heterogeneity led to a misdiagnosis of primary intrahepatic cholangiocarcinoma at another hospital. Molecular analysis showed that both lung and liver tumors harbored consistent *SMARCA4* and *TP53* mutations. Clonal evolutionary analysis revealed that both major and subclones were more prevalent in the lung tumor than in the liver metastasis. The evolutionary tree topology strongly suggested a unidirectional trajectory from the primary lung tumor to liver metastasis.

**Conclusion:**

This study provides the first genomic evidence of shared and divergent somatic mutations in *SMARCA4*-dNSCLC and its hepatic metastasis. Clonal evolutionary analysis confirmed the diagnosis of *SMARCA4*-dNSCLC with hepatic metastasis, resolving diagnostic challenges and supporting precision therapy.

## Graphical Abstract

**Figure:**
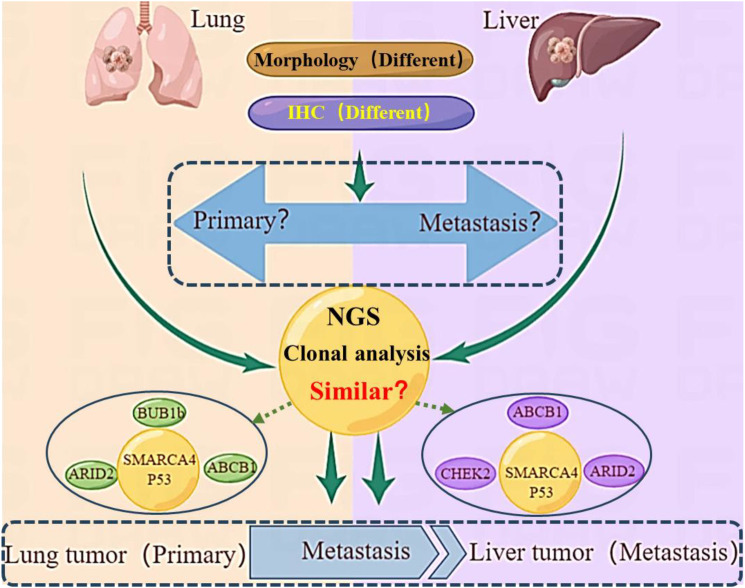

## Introduction

*SMARCA4*-deficient non-small cell lung cancer (*SMARCA4*-dNSCLC) accounts for at least 10% of all non-small cell lung cancers (NSCLCs) (1). This cancer has complex clinical manifestations and pathological characteristics, including high malignancy, histomorphological heterogeneity, multiple genetic drivers, rapid tumor progression, and poor rates of survival (2, 3), which accordingly pose challenges in gaining a requisite clinical understanding. *SMARCA4*-dNSCLC often harbors co-occurring mutations in *SMARCA4*, *KRAS*, *TP53*, *KEAP1*, and *STK11*, whereas common driver mutations in *ALK*, *EGFR*, and *ROS1* are rarely observed (4, 5). Conventional therapies, such as radiotherapy, chemotherapy, and targeted treatments, have not significantly improved the prognosis for patients with *SMARCA4*-dNSCLC. Metastasis, particularly to distant organs, is a primary cause of cancer-related mortality. Diagnosing *SMARCA4*-dNSCLC accurately based on histomorphology and immunohistochemistry is challenging, as these tumors generally lack the expression of lung lineage markers, such as Napsin A and TTF-1. This diagnostic dilemma is exacerbated when *SMARCA4*-dNSCLC co-occurs with a hepatic tumor, as there are currently no reliable methods that can be used to definitively determine whether the primary tumor is derived from the lungs or liver.

Although tumor cell heterogeneity is a common characteristic of cancer, the biological significance of heterogeneous tumor clones remains poorly understood. Most tumors comprise heterogeneous cell populations, and during tumor progression, genomic instability and variations in the tumor microenvironment can induce distinct genetic and epigenetic changes in tumor clones. Notably, however, primary tumor growth is associated with a reduction in clonal heterogeneity (6, 7), although the clonal alterations that drive distant metastasis during primary tumor progression have yet to be sufficiently determined. This accordingly highlights the need for a more comprehensive understanding of clonal composition and evolution in tumor progression and metastatic dissemination, facilitating more accurate diagnoses and developing novel therapeutic strategies.

Mutations in *SWI/SNF* complex subunits are widely distributed among different malignancies, with alterations in the *SMARCA4* subunit (BRG-1) being identified as the most frequent (8, 9). In NSCLC, mutations in*SMARCA4*, the rates of which can range from 8% to 11%, can serve as independent prognostic factors associated with increased tumor aggressiveness and poor prognosis (10). The distinct morphological and genetic profiles of *SMARCA4*-dNSCLC define it as a unique subtype that accordingly requires comprehensive diagnostic evaluation (11). Patients with *SMARCA4*-dNSCLC typically present at a median age of approximately 60 years, with a male predominance, and many have a history of long-term tobacco use (11). Early symptoms are often nonspecific and may include dyspnea, pain, cough, and hemoptysis. The clinical presentation and pathological features of *SMARCA4*-dNSCLC are complex, characterized by significant intertumoral heterogeneity, histomorphological diversity, and the involvement of multiple genetic drivers. These factors pose substantial challenges for the accurate clinical characterization of this disease (12). In addition, *SMARCA4*-dNSCLC tends to be characterized by poor responsiveness to conventional chemotherapy and lacks common targetable driver mutations. Consequently, there is currently a distinct lack of effective targeted therapies for the treatment of this subtype (13). Accordingly, the accurate identification of these tumors, along with the development of more precise and effective therapeutic strategies to improve patient quality of life and survival outcomes, remains an urgent clinical priority.

*SMARCA4*-dNSCLC is characterized by diverse histological patterns, most commonly manifesting as a poorly differentiated type of NSCLC. Microscopically, tumor cells are typically arranged in solid sheets and nests with focal glandular formations. These cells display significant atypia, predominantly characterized by an epithelioid morphology. Nuclei are vesicular with prominent nucleoli, and numerous mitotic figures and geographic necrosis are typically observed. *SMARCA4*-dNSCLC often requires differential diagnosis from other poorly differentiated neoplasms, including *SMARCA4*-deficient undifferentiated tumors, large cell neuroendocrine carcinoma, NUT carcinoma, melanoma, germ cell tumors, and metastatic *SMARCA4*-deficient tumors. Genomically, *SMARCA4*-dNSCLC is characterized by distinct genetic alterations, including mutations in *STK11*, *KEAP1*, and *KRAS*, which distinguish it from common drivers of lung adenocarcinoma. The predominant genetic alteration is the *KRAS* mutation (14). Immunohistochemically, *SMARCA4*-dNSCLC is typically characterized by the loss of both BRG-1 and TTF1 expression, although in a small subset of cases, the expression of TTF1 is retained. The cells are frequently positive for CK7 and HepPar-1, the expression of the latter of which is often diffuse and strong, with a characteristic granular (mitochondrial) cytoplasmic pattern reminiscent of hepatocellular carcinoma (15, 16). The diagnostic challenges are particularly pronounced in circumstances in which *SMARCA4*-dNSCLC occurs synchronously with poorly differentiated hepatic tumors. In such cases, distinguishing between metastatic *SMARCA4*-dNSCLC and liver tumors, synchronous primary *SMARCA4*-dNSCLC with a separate *SMARCA4*-deficient hepatic tumor, or a metastatic *SMARCA4*-deficient hepatic tumor in the lungs, can prove exceedingly difficult based solely on histomorphology, immunohistochemistry, and limited molecular testing.

This study employed a multifaceted approach that extended beyond conventional histopathological morphology and immunophenotypic analyses. We prioritized somatic mutation detection in both pulmonary and hepatic tumors. This research directly addresses the critical diagnostic challenge of determining tumor origin. By providing a robust method for accurately identifying primary tumors and their metastases, our findings introduce a novel molecular approach. This lays the foundation for improved diagnostic accuracy and facilitates the development of precise therapeutic strategies for patients with *SMARCA4*-dNSCLC and concurrent tumors in other organs.

## Materials and methods

### Study design and subjects

The retrospective study included patients recruited at the Xiamen Humanity Hospital between December 2019 and May 2025. The study was approved by the ethical committee of Xiamen Humanity Hospital (HAXM-MEC-20250814-064-01). All included subjects signed an informed consent. The study protocol was in accordance with the Declaration of Helsinki and was approved by the Institutional Review Board of Xiamen Humanity Hospital.

### Clinicopathologic data

In March 2019, a 60-year-old patient underwent surgical resection for an adenocarcinoma of the left upper lobe (pT1cN2M0, Stage IIIA). From April to July 2019, the patient completed four cycles of adjuvant chemotherapy with pemetrexed plus carboplatin, followed by postoperative adjuvant radiotherapy (total dose 50 Gy) from July to August 2019. Subsequently, the patient entered a period of regular follow-up without further antitumor therapy. The follow-up was performed every 3 months, including chest CT, abdominal color Doppler ultrasound and serum CEA detection, all of which were negative. After a disease-free interval of approximately 44 months, in November 2022, an abdominal Magnetic Resonance Imaging(MRI)revealed a metastatic lesion in segment IV of the liver, along with multiple hemangiomas in both lobes, and chronic cholecystitis with cholelithiasis. Subsequent abdominal Computed Tomography (CT) identified a hypodense lesion in segment IV, which was suspected to be indicative of metastasis. The patient subsequently underwent laparoscopic partial hepatectomy for the metastatic lesion with concomitant cholecystectomy. Based on histopathological examination of the surgical specimen at an external institution, the hepatic lesion was diagnosed as a primary intrahepatic cholangiocarcinoma.

### Hematoxylin and eosin staining

We immersed the tumor tissues in 10% paraformaldehyde at 4°C overnight. After sequential dehydration in ethanol and xylene, the tissue was embedded in paraffin. Having subsequently removed the paraffin wax with xylene, the samples were rehydrated using a high- to low-concentration ethanol gradient. The sections were subsequently stained with hematoxylin for 5 min and with eosin for 6min.

### Immunohistochemical staining

Deparaffinized liver and lung tumor sections were fixed in paraformaldehyde and subsequently incubated overnight at 4°C with the following primary antibodies: TTF-1(Clone: MX011), Napsin A(Clone: MX015), CK7(Clone: MX053), Heppar-1(Clone: MAB-1034), Glypican-3(Clone: MAB-0667), CD34(Clone: MX123), PD-L1 (Clone:22C3) CK19(Clone: MAB-0829), CK20(Clone: MAB-0834), GS(Clone: MAB-0752), p53(Clone: MC008), BRG-1 (Clone: RMA-1063), Ki67(Clone: SP6), HBcAg(Clone: MAB-0899), D2-40(Clone: MAB-0567), SALL4(Clone: MAB-1093), and CD56(Clone: MX039). Tissues were incubated overnight at 4°C with the antibodies. A secondary antibody was then added to sections, followed by incubation at 25°C for one hour. Then, wash sections repeatedly with PBS buffer, add the chromogenic reagent DAB (Lot: DAB-0031, Fuzhou Maixin biotech.Co., Ltd.,China), and generate brown precipitate through enzyme-catalyzed reaction, then terminate the reaction. Observe the results under an optical microscope and take photos to record the experimental data. All antibodies were purchased from Fuzhou Maixin biotech.Co.Ltd., China and ZSGB-BIO, Co.Ltd,China.

### Next-generation sequencing

#### DNA extraction and library construction

Genomic DNA was extracted from FFPE samples using a QIAamp DNA FFPE Tissue Kit (Qiagen). Sequencing libraries were prepared utilizing a KAPA Hyper Prep Kit (KAPA Biosystems) in accordance with the manufacturer’s guidelines. The DNA libraries thus obtained were then PCR amplified, and the resulting products were purified using Agencourt AMPure XP beads. Customized xGen lockdown probes targeting 437 cancer-relevant genes were used for hybridization enrichment.

#### Sequencing and bioinformatics analysis

Targeted enriched libraries were sequenced using a NovaSeq 6000 sequencer (Illumina) with 2× 150 bp paired-end reads. Single-nucleotide variants (SNVs) and indels were identified using VarScan2 and the HaplotypeCaller/UnifiedGenotyper in GATK, with a mutant allele frequency (MAF) threshold of 1%. Gene fusions were identified via FACTERA and copy number variations (CNVs) were analyzed using CNV Kit. The log2 ratio threshold for copy number gains in tissue samples was set at 2.0, whereas a threshold of 0.6 was applied for copy number losses. The tumor mutational burden (TMB) was defined as the number of somatic, non-synonymous, and indel mutations per megabase of the genome examined.

### Clonal evolution

Clonal fractions for each sample were calculated using the Pyclone tool (17), whereas SCHiSM was used to facilitate prediction of subclonal hierarchy and evolutionary relationships (18). Fish plots were drawn using Timescape (v3.14).

### Statement

Our work has been reported in line with the ARRIVE guidelines 2.0.

## Results

### Imaging findings

**Figure 1:**
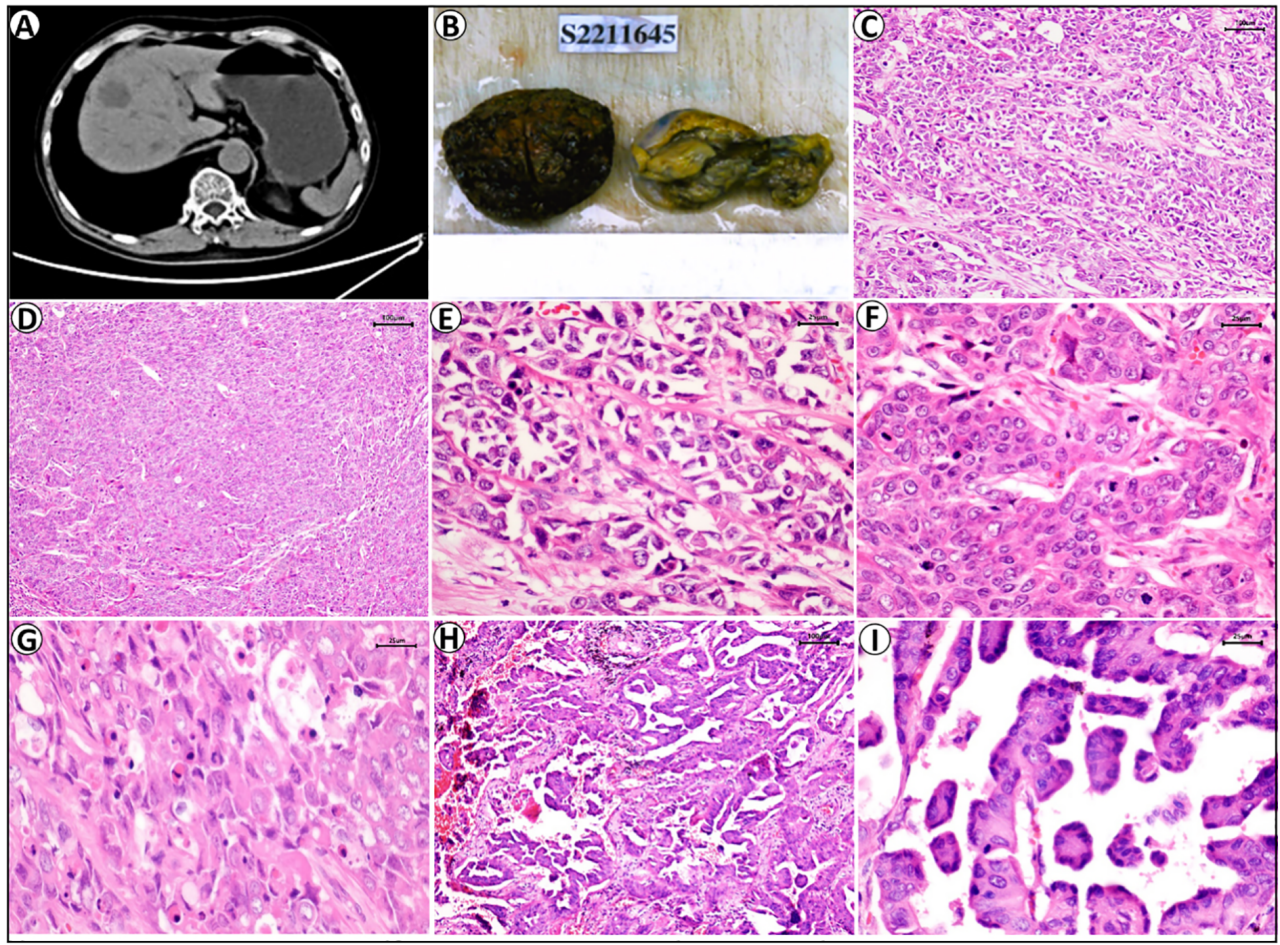
The histological morphology and imaging examination of liver tumors and lung tumors. **(A)** Concurrent abdominal CT demonstrated a hypodense lesion in segment IV of liver. **(B)** Gross specimens of whole liver tumor and gallbladder. **(C, D)** HE staining showed liver tumor cells were arranged in focal loss of cohesion, solid nests, cords, and acinar structures (HE, 100×); **(E-G)** High-power examination demonstrated significant cellular atypia, prominent nucleoli, frequent mitotic figures, and focal areas showed eccentrically placed nuclei imparting a rhabdoid morphology in liver tumor (HE, 400×). **(H, I)** The pulmonary tumor cells were arranged in acinar and micropapillary patterns, with marked nuclear atypia. (HE, 100× 400×).

Abdominal MRI performed in 2022 revealed the presence of a metastatic lesion in segment IV of the liver, accompanied by multiple hemangiomas in both hepatic lobes, along with chronic cholecystitis and multiple gallstones. Concurrent abdominal CT revealed a hypodense lesion in segment IV, which was considered to be indicative of metastasis (**Figure 1A**).

### Gross view of tumor

The resected hepatic specimen measured 7.4 × 6.0 × 3.2 cm. Sectioning revealed a well-demarcated tumor mass measuring 3.2 × 2.5 × 2.4 cm. The cut surface of the tumor was homogeneously gray white in color and moderately firm. Based on a gross examination, we failed to identify any satellite nodules and obtained no evidence to indicate lymphovascular invasion (**Figure 1B**).

### Histopathological morphology

(Liver tumor): At low-power magnification, the neoplastic cells were observed to be characterized by an epithelioid morphology and were arranged in solid nests, cords, acinar structures, and glandular formations, with focal loss of cohesion and associated stromal fibrosis. High-power examination enabled us to identify significant cellular atypia, prominent nucleoli, and frequent mitotic figures. The tumor cells were observed to have an eosinophilic cytoplasm, and focal areas were characterized by eccentrically distributed nuclei, imparting a rhabdoid morphology (**Figures 1C–G**).

(Lung tumor): The lung tumors were adenocarcinomas, comprising 70% alveolar adenocarcinomas and 30% micropapillary adenocarcinomas (**Figures 1H, I**).

### Immunohistochemical analysis

**Figure 2:**
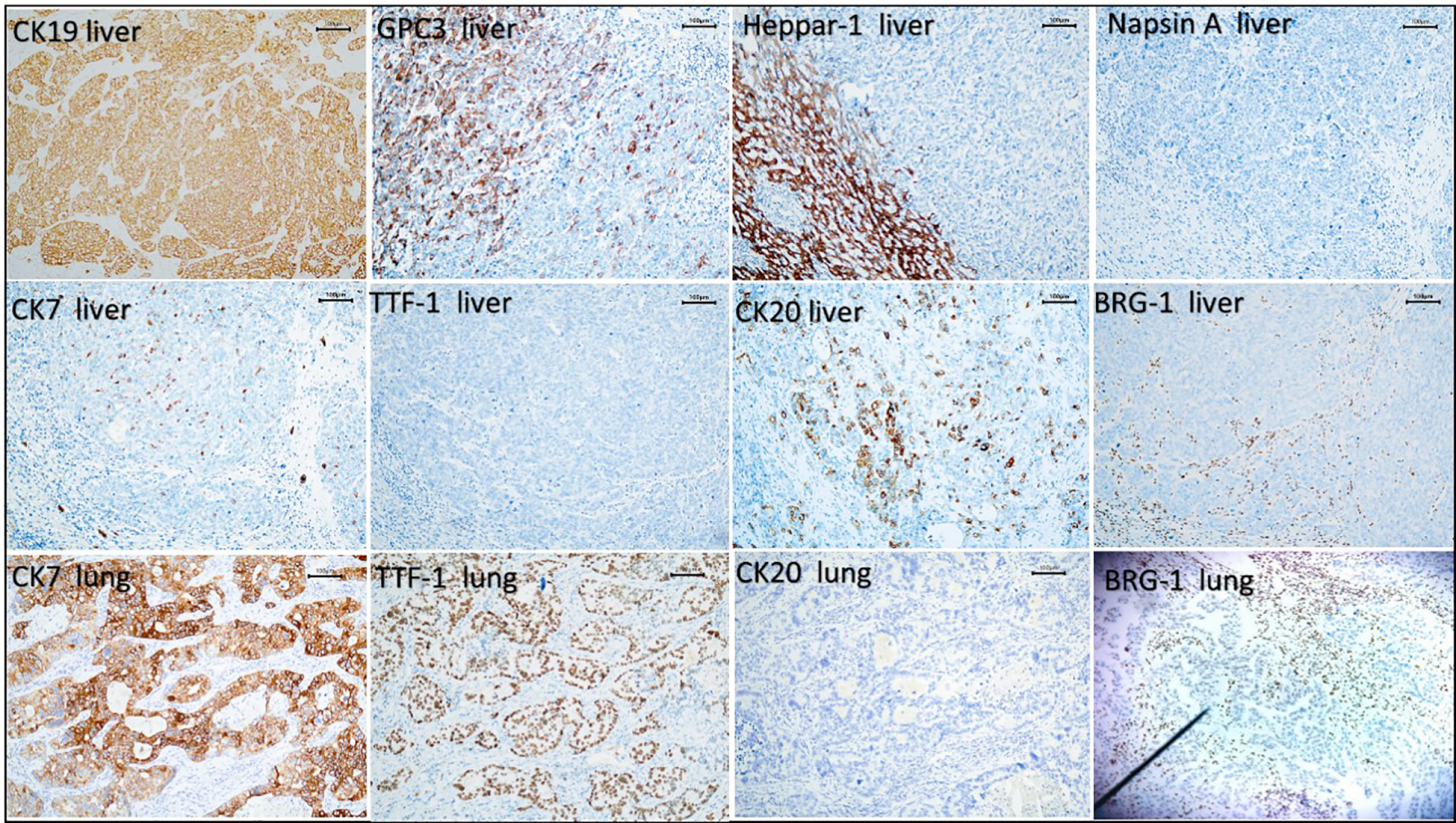
The immunohistochemical results showed that the liver tumor expressed CK19, focal expressed CK20, CK7 and GPC3, and did not express HepPar-1, Napsin-A,TTF-1, or BRG-1. Lung tumors expressed CK7 and TTF-1,did not express CK20 or BRG-1 (100×).

In terms of immunophenotype, we established that the liver tumor expressed CK19 and GS, focal expressed CK7, Glypican-3, and CK20, although not p53, BRG-1, CD34, HepPar-1, HBcAg, D2-40, CD56, TTF-1, Napsin A, or SALL4, and we obtained a proliferation index (Ki-67) value of approximately 70%. In comparison, whereas lung tumors expressed CK7 and TTF-1, we detected no expression of BRG-1, CK20, Napsin A, CD56, CgA, Syn, CK5/6, p63 or Glypican-3, and found that the proliferation index value was approximately 40% (**Figure 2**).

### Next-generation sequencing

**Figure 3:**
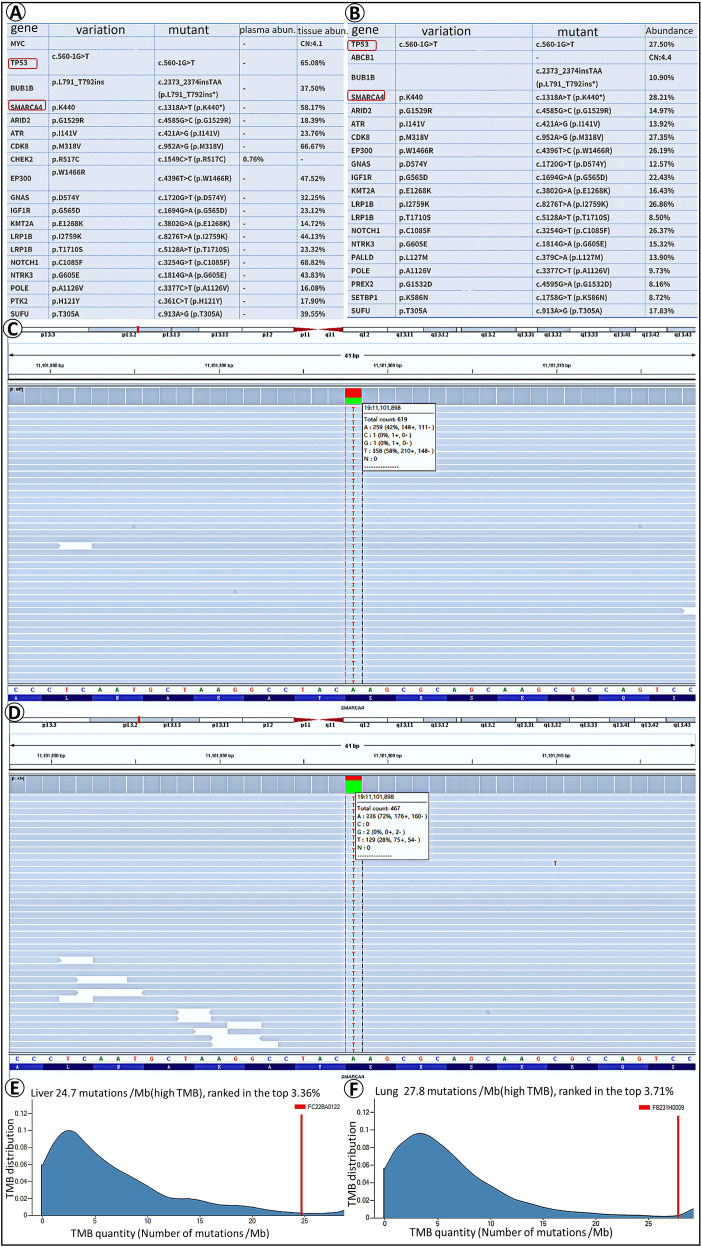
Next-generation sequencing analysis. Results showing *P53* and *SMARCA4* mutation in liver **(A)** and lung **(B)** tumor. The mutation profiles of SMARCA4 in the hepatic metastasis **(C)** and primary pulmonary lesion **(D)** are presented, respectively. The gene mutation burden in liver tumors is 24.7 mutations/Mb (high TMB) **(E)**, and the gene mutation burden in lung tumors is 27.8 mutations/Mb (high TMB) **(F)**.

Analysis based on next-generation sequencing revealed identical somatic mutations in both the liver (**Figure 3A**) and lung (**Figure 3B**) tumors, including a *TP53* splice-site mutation (c.560-1G>T, intron 5) and a *SMARCA4* nonsense mutation (c.1318A>T, p.Lys440*, exon 8). The mutation profiles of SMARCA4 in the liver metastatic tumor and primary lung lesions are presented (**Figures 3C, D**). For the liver tumor, we obtained a tumor mutational burden (TMB) value of 24.7 mutations/Mb (high TMB) (**Figure 3E**), and a corresponding value of 27.8 mutations/Mb (high TMB) for lung tumors (**Figure 3F**). These results accordingly revealed concordant driver gene mutations in the lung and liver tumors, thereby providing molecular evidence in support of their clonal relatedness.

### Clonal evolution

**Figure 4:**
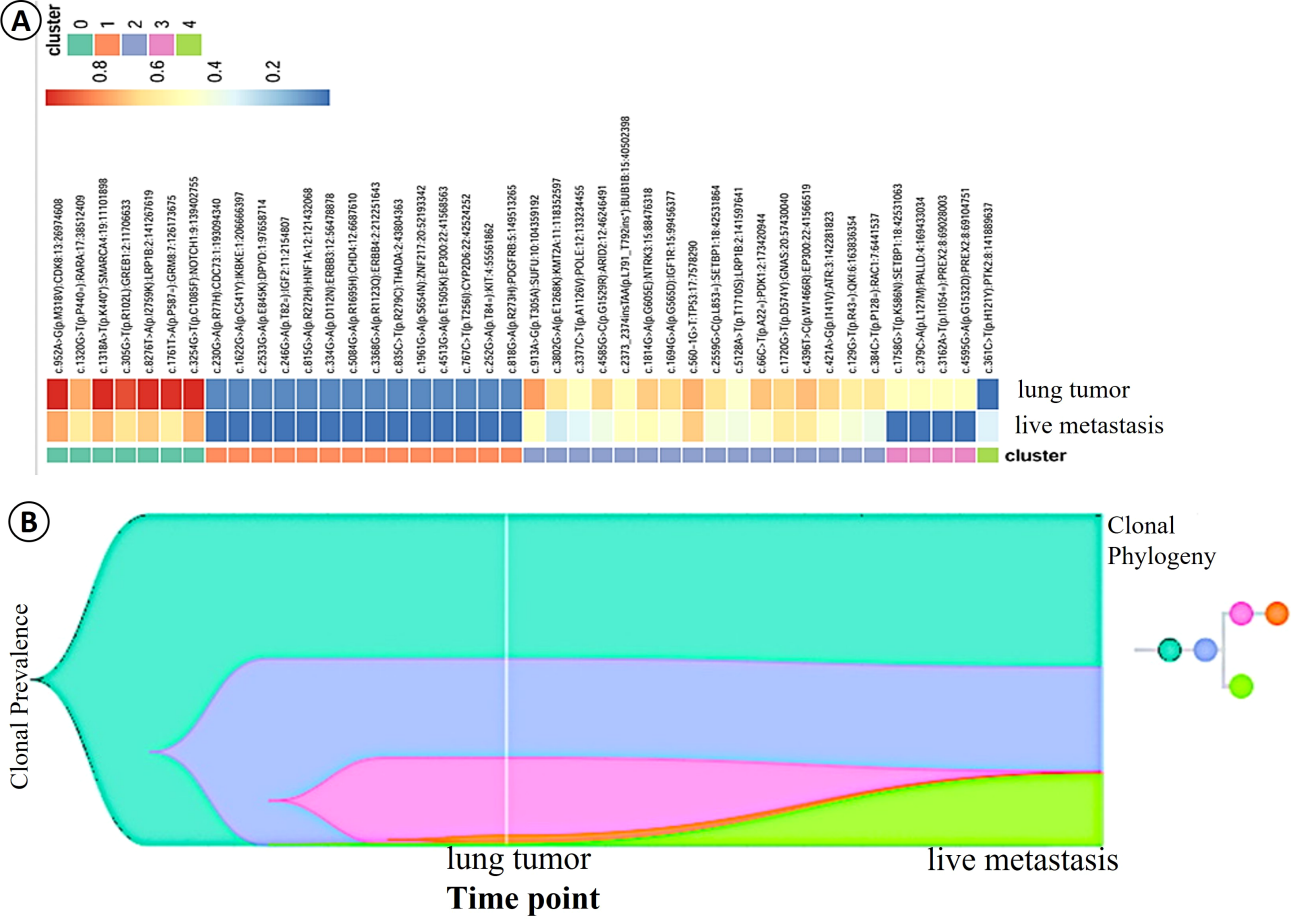
Analysis of clonal evolution and Fish plots. **(A)** Compared with the hepatic metastatic sample (FC22BA0122), the founding clone (clone 0) and a major subclone (clone 2) were established to have a higher clonal prevalence within the lung tumor sample (FB231H0009). **(B)** The inferred phylogenetic relationship, based on the evolutionary tree topology, provided convincing evidence to indicate a unidirectional evolutionary trajectory from the primary lung tumor to the hepatic metastatic tumor.

Clonal evolutionary analysis revealed that *SMARCA4* mutations, assessed based on the cancer cell fraction (CCF; also referred to as cellular prevalence, CP), were predominantly enriched in the lung lesions (CCF: 98.36%) and significantly less prevalent in the liver lesions (CCF: 72.89%). Furthermore, compared with the liver metastatic sample (FC22BA0122), the founding clone (clone 0) and a major subclone (clone 2) were established to have a higher clonal prevalence within the lung tumor sample (FB231H0009). Whereas clones 0 and 2 were present in both lesion types, subclones 1 and 3 were predominantly detected in the lung lesions, and a distinct subclone (clone 4) was found to be predominant in the liver lesion (**Figure 4A**). The inferred phylogenetic relationship, based on the evolutionary tree topology, provided convincing evidence to indicate a unidirectional evolutionary trajectory from the primary lung tumor (FB231H0009) to the liver metastatic tumor (FC22BA0122) (**Figure 4B**). This clonal analysis thus provides definitive molecular evidence to identify the hepatic lesion as a metastatic tumor originating from the primary *SMARCA4*-deficient pulmonary adenocarcinoma.

### PD-L1 testing results in hepatic tumor

**Figure 5:**
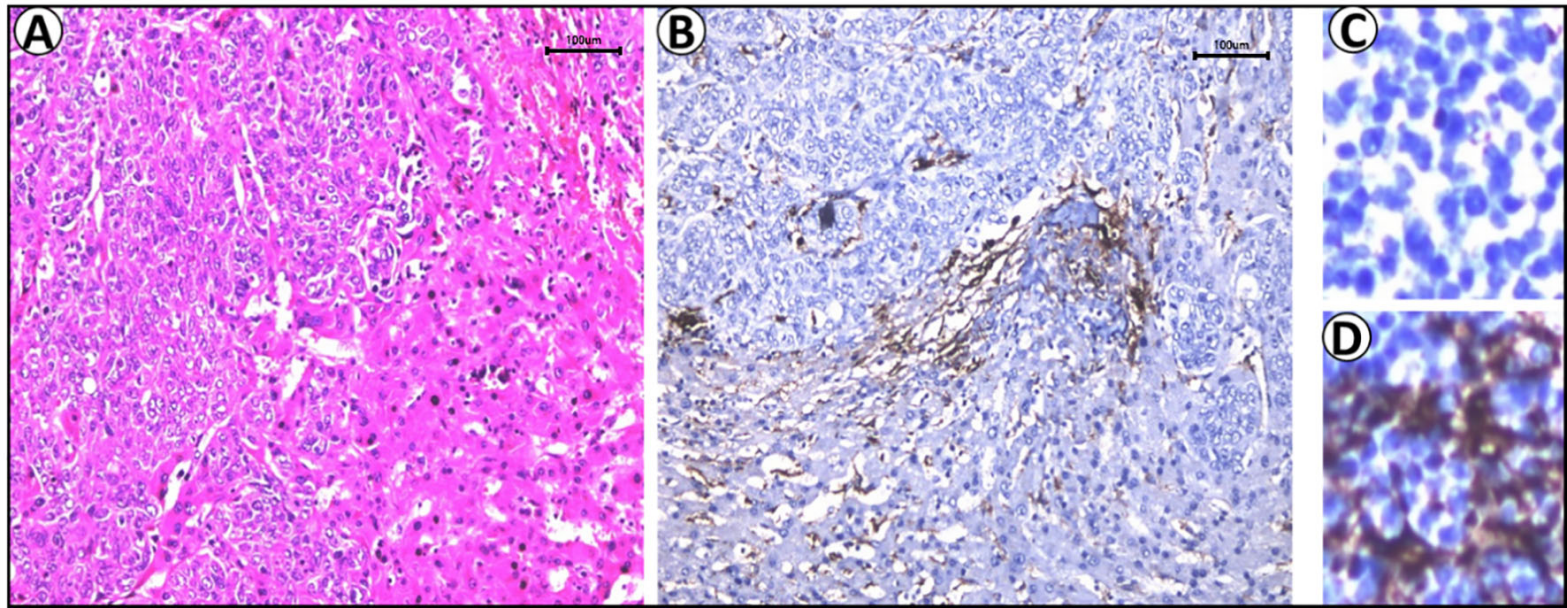
PD-L1 testing result in Hepatic Tumor. **(A)** The tissue morphology of liver tumors by HE staining. **(B)** PD-L1 Expression by TPS: Negative (TPS <1%). **(C)** Negative control. **(D)** Positive control.

PD-L1 Expression by TPS: Negative (TPS <1%); PD-L1 Expression by CPS: Positive (CPS ≥1; CPS = 2) (**Figure 5**). Despite a negative PD-L1 testing result, immunotherapy was initiated as second-line therapy based on the patient’s clinical circumstances and with consent from the patient.

## Discussion

*SMARCA4*-dNSCLC is characterized by complex and heterogeneous clinical manifestations and pathological features. Distant metastases often represent the initial clinical presentation (19, 20). This tumor type lacks specific immune markers that are typically used to identify the tissue of origin presents a substantial diagnostic challenge in situations in which *SMARCA4*-dNSCLC co-occurs with a hepatic tumor (21, 22). Some case reports have indicated that the deletion of *SMARCA4* can occur in intrahepatic cholangiocarcinomas (23) and undifferentiated hepatic carcinomas (24). This accordingly highlights the necessity of gaining a more comprehensive understanding of clonal composition and evolution during tumor progression and metastatic dissemination. In this study, we addressed the critical question regarding the mutational concordance between pulmonary and hepatic lesions and accordingly confirmed that *SMARCA4*-dNSCLC is the primary tumor, whereas the hepatic lesion is a metastatic derivative. Elucidating this relationship is essential for accurate tumor identification, gaining an understanding of intra-tumoral heterogeneity, and enabling more precise therapeutic strategies for affected patients.

The SMARCA4 p.K440* mutation identified in this study was functionally categorized as a Class 1 (clearly pathogenic) variant leading to protein truncation in the study by Fernando TM et al. (25), confirming its role as a core driver. We performed an analysis using public data from cBioPortal. By comparing the mutation profile of the liver metastasis in this study with the mutational population characteristics of SMARCA4-mutant hepatocellular carcinoma (n=80, https://bit.ly/4sFvygc) and lung adenocarcinoma (n=438, https://bit.ly/4qvs4LJ), we found that: ① No mutations were detected in the most common series of characteristic genes (e.g., TERT, CDKN2A, AXIN1, etc., with mutation frequencies >20% in that cohort) other than TP53 that are typical of SMARCA4-mutant hepatocellular carcinoma; ② This study harbored the characteristic high-frequency co-mutated gene LRP1B found in SMARCA4-mutant lung adenocarcinoma (frequency in the lung adenocarcinoma cohort: 51.55%; frequency in the hepatocellular carcinoma cohort: 15.63%). In summary, from the perspective of population-level molecular characteristics, the genomic profile of the liver lesion in this study is highly similar to that of lung adenocarcinoma, yet distinct from primary hepatocellular carcinoma. This finding provides population-level data in addition to clonal evolution analysis, supporting the conclusion that the hepatic lesion originated from a pulmonary source.

Notably, significant morphological and immunophenotypic heterogeneity was observed between the primary lung tumor and its hepatic metastasis. Morphologically, the lung lesion predominantly exhibited acinar and micropapillary patterns, whereas the liver metastasis demonstrated a primarily solid architecture with discohesive tumor cells and focal rhabdoid features. Immunophenotypically, the primary tumor showed diffuse CK7 and TTF1 expression without CK20 expression, while the metastatic lesion displayed attenuated CK7 expression, loss of TTF1, and focal CK20 positivity. These phenotypic divergences compounded the diagnostic challenge of distinguishing *SMARCA4*-dNSCLC metastasis to the liver from synchronous primary pulmonary and hepatic tumors. This diagnostic difficulty led the referring institution to misclassify the hepatic lesions as intrahepatic cholangiocarcinomas. As a result, liver metastasis from *SMARCA4*-deficient lung adenocarcinoma (initially diagnosed two years prior as pT1cN2M0, Stage IIIA; now progressing to hepatic involvement with current clinical Stage IV) was erroneously interpreted as primary intrahepatic cholangiocarcinoma (pT1aN0M0, Stage IA), leading to a significant underestimation of the patient’s true disease burden. This diagnostic error resulted in the misclassification of advanced metastatic lung cancer (Stage IV) as an early-stage localized hepatobiliary malignancy (Stage IA), creating a three-tier discrepancy in disease staging. The direct consequence was a misalignment in the therapeutic strategy. Instead of receiving systemic therapies for advanced lung cancer (e.g., platinum-based chemotherapy, targeted agents, or immune checkpoint inhibitors), the patient was potentially diverted to locoregional treatments appropriate for early-stage cholangiocarcinoma (e.g., surgical resection). Such mismanagement not only accelerates disease progression due to undertreatment but also generates unrealistically optimistic prognostic assessments based on erroneous staging, ultimately compromising overall survival. Despite the revised diagnosis at our institution and prompt initiation of tislelizumab plus pemetrexed-cisplatin therapy, the patient experienced disease progression within six months. This further supports the notion that delayed recognition of metastatic lung cancer significantly compromises therapeutic responsiveness.

It should be emphasized that the clinical course (44-month disease-free interval followed by hepatic lesion) can only provide a preliminary clue for metastasis but cannot exclude the possibility of double primary tumors. Clonal evolution analysis, as a gold standard for confirming clonal relatedness, not only verified that the two lesions originated from the same clone but also revealed the evolutionary process of tumor metastasis, which is crucial for correcting misdiagnosis, determining accurate tumor staging, and guiding subsequent treatment. Without clonal evolution analysis, the misclassification of advanced metastatic lung cancer as early-stage intrahepatic cholangiocarcinoma would persist, leading to inappropriate treatment and poor patient prognosis. Therefore, exploring somatic mutations in *SMARCA4*-dNSCLC and liver tumors, analyzing subclonal evolution in these tumors, and distinguishing the primary lesion from the metastatic lesion are crucial.

A recently published study by Duo Xie et al. reported that most clonal driver mutations are shared between primary tumor–metastasis pairs (72% for brain metastases, 79% for extracranial metastases, and 89% for lymph node metastases) (26). Our results demonstrated a high degree of concordance between the mutational spectra of both tumors. Specifically, sixteen of the twenty mutations detected in the primary lung lesion were also identified in the liver lesion, including identical core driver mutations (*SMARCA4* and *TP53*), confirming their clonal relatedness. This analysis conclusively identified the lung tumor as the primary lesion and the liver tumor as the metastatic deposit. This study resolves a significant diagnostic dilemma and provides crucial evidence for accurately determining the tumor origin. These findings establish a crucial foundation for targeted oncology treatments with substantial clinical implications.

However, our study has several limitations. Although this study provides a novel molecular approach for determining tumor origin, the relatively small sample size limits our ability to definitively establish whether subclones are present in all *SMARCA4*-dNSCLC patients with hepatic metastases. Future studies will focus on gathering more comprehensive clinicopathological data from a larger cohort of *SMARCA4*-dNSCLC patients with hepatic metastases. This expanded investigation will allow for a more thorough exploration of the clonal architecture and evolution of these tumors. Ultimately, we aim to contribute to the development of more precise diagnostic and therapeutic strategies, thereby improving patient prognosis.

The innovation of this study lies not only in the first systematic application of clonal evolution analysis for the differential diagnosis of SMARCA4-dNSCLC metastases, thereby deepening the understanding of the metastatic mechanisms of this tumor, but also in proposing a generalizable new model of “molecular pathology combined with evolutionary analysis”. This model effectively addresses a long-standing diagnostic blind spot in clinical practice and holds significant academic value and potential for clinical translation.

## Conclusion

In this study, we conducted a detailed exploration of pulmonary and hepatic tumors through histopathological, immunophenotypic, and molecular analyses. For the first time, we utilized comprehensive genetic profiling to elucidate the similarities and differences in somatic mutations between pulmonary and hepatic lesions. Furthermore, clonal evolution analysis confirmed that *SMARCA4*-dNSCLC was the primary tumor, with the hepatic lesion identified as a metastatic derivative. These findings bridge a significant gap in current research, which has been primarily theoretical, and provide novel molecular evidence to guide personalized, precise diagnosis and treatment for patients with this complex presentation.

## Data Availability

The original contributions presented in the study are included in the article/**Supplementary Material**. Further inquiries can be directed to the corresponding author.
