# Supplementary material for: Therapeutic misalignment averted by clonal evolutionary evidence: molecular confirmation of hepatic metastasis in SMARCA4-deficient non-small cell lung cancer initially misdiagnosed as resectable cholangiocarcinoma
Source: Front Oncol. 2026 Mar 11;16:1743908. doi: 10.3389/fonc.2026.1743908 (PMC13012966; doi:10.3389/fonc.2026.1743908)
Supplement: Supplementary file 1 [file DataSheet1.docx]

**Supplementary Materials**

**1. Detailed Explanation Regarding the Selection of Representative Tumor Regions:**

This study strictly adhered to standardized procedures for sample selection in tumor genomic analysis. **(1) For the primary lung lesion**, as the surgery was performed at an external institution, the FFPE block we obtained contained tumor tissue that had been clearly identified in the external pathological diagnosis. The tumor area in consecutive sections from this block measured 1.6 cm × 1.4 cm. After review by two senior pathologists, it was confirmed that the sample fully encompassed the two main growth patterns—acinar and micropapillary—described in the original pathology report. Therefore, this sample is considered to possess adequate histological representativeness. **(2) For the hepatic metastasis** (3.2 × 2.5 × 2.4 cm), a systematic histological assessment of the entire tumor was conducted. During the preparation of the FFPE tissue block for NGS, a section was deliberately selected to maximize representation of the tumor's overall heterogeneity. This section consisted of approximately 55% solid/acinar architecture and about 45% area exhibiting loss of cellular cohesion accompanied by stromal reaction (fibrosis). This composition was largely consistent with the evaluation of the entire gross tumor specimen, thereby ensuring that the sampled tissue was representative of the tumor's overall cellular composition.

**(3) Assessment of Tumor Purity (Cellularity):** During the NGS sample preparation stage, consecutive sections were cut from the aforementioned FFPE block. The first paraffin section was stained with hematoxylin and eosin (H&E) and dedicated specifically for pathological quality control. The evaluation of tumor cellularity was independently performed by two senior pathologists under light microscopy, following the assessment rules outlined below:

① **Definition:** Tumor cellularity is defined as the percentage of tumor cell nuclei relative to all nucleated cells, including tumor cells, lymphocytes, fibroblasts, and others.

② **Method:** The entire slide was scanned at low-power magnification (×40 or ×100). Areas with obvious necrosis, hemorrhage, or pure stroma were excluded. Several of the most representative fields of view were then selected and visually estimated at high-power magnification (×200 or ×400), and the average value was calculated.

③ **Results:** The difference between the two assessors was less than 10%, and the average was taken as the final reported value. The estimated tumor cellularity was approximately 30% for the primary lung lesion and approximately 70% for the hepatic metastasis. The significantly higher purity in the liver lesion was primarily attributed to the high proportion of solid tumor cell nests in the sampled region, whereas the cellular components within areas of stromal reaction (fibrosis) were relatively sparse.

**2. Regarding the accuracy of our clonal architecture reconstruction, we provide the following detailed explanation:**

Our clonal analysis explicitly incorporated both copy number variation (CNV) data and sample-specific purity estimates. While CNVs themselves were not treated as independent subclonal events in our pipeline, the local copy number information was used to correct variant allele frequencies (VAFs) of somatic SNVs to accurately determine their cellular prevalence (CP). The detailed workflow and parameters are as follows:

**Data Preparation:** For this patient, we used longitudinal sampling at two time points. The analysis targeted somatic SNVs, excluding germline mutations with a paired control. We used an automated bioinformatics pipeline to generate three essential inputs for clonal analysis: **somatic mutations, CNVs, and sample-specific tumor purity estimates**.

**Clonal Clustering (PyClone):** Clonal architecture was inferred using PyClone, which models relationships between read counts, tumor purity, and local copy numbers. Inputs included r**eference and variant read counts, sample-specific purity, and CNV date (minor_cn/major_cn/normal_cn).** PyClone infers Cellular Prevalence (CP) of mutations by accounting for confounding effects of purity and copy number alterations.

**Phylogenetic Tree Reconstruction (SCHISM):** SCHISM was used to reconstruct evolutionary history following cluster identification. It uses a Genetic Algorithm (GA) to build phylogenetic trees from PyClone-derived CP values. It constructs a hypothesis test to obtain a cellular prevalence violation (CPOV) matrix and optimizes tree topology using a fitness function incorporating lineage precedence and divergence rules.

**Visualization (Timescape):** Evolutionary trajectories and clonal dynamics were visualized as “fish plots” using Timescape, providing an intuitive view of subclonal expansion and contraction over time.
